# Association of drug overdoses and user characteristics of Canada’s national mobile/virtual overdose response hotline: the National Overdose Response Service (NORS)

**DOI:** 10.1186/s12889-023-16751-z

**Published:** 2023-09-27

**Authors:** Dylan Viste, William Rioux, Nora Cristall, Taylor Orr, Pamela Taplay, Lisa Morris-Miller, S. Monty Ghosh

**Affiliations:** 1 Department of Medicine, Cumming School of Medicine, University of Calgary, Calgary, AB Canada; 2 Department of Medicine, Faculty of Medicine & Dentistry, University of Alberta, Edmonton, AB Canada; 3 Centre for Health Informatics, University of Calgary, Calgary, AB Canada; 4 Grenfell Ministries, Hamilton Ontario, Canada; 5 Department of Internal Medicine, Faculty of Medicine and Dentistry, University of Alberta, Edmonton, AB Canada

**Keywords:** Overdose, Harm Reduction, Public Health, Telehealth, Supervised Consumption Services, Virtual overdose prevention services, Mobile overdose response services, MORS

## Abstract

**Background:**

Several novel overdose response technology interventions, also known as mobile overdose response services (MORS), have emerged as adjunct measures to reduce the harms associated with the drug poisoning epidemic. This retrospective observational study aims to identify the characteristics and outcomes of individuals utilizing one such service, the National Overdose Response Service (NORS).

**Methods:**

A retrospective analysis was conducted using NORS call logs from December 2020 to April 2023 imputed by operators. A variety of variables were examined including demographics, substance use and route, location, and call outcomes. Odds ratios and 95% confidence intervals were calculated around variables of interest to test the association between key indicators and drug poisonings.

**Results:**

Of the 6528 completed calls on the line, 3994 (61.2%) were for supervised drug consumption, 1703 (26.1%) were for mental health support, 354 (5.42%) were for harm reduction education or resources, and 477 (7.31%) were for other purposes. Overall, there were 77 (1.18%) overdose events requiring a physical/ in-person intervention. Of the total calls, 3235 (49.5%) were from women, and 1070 (16.3%) were from people who identified as gender diverse. Calls mostly originated from urban locations (*n* = 5796, 88.7%) and the province of Ontario (*n* = 4137, 63.3%). Odds ratios indicate that using opioids (OR 6.72, CI 95% 3.69–13.52), opioids in combination with methamphetamine (OR 9.70, CI 95% 3.24–23.06), multiple consumption routes (OR 6.54, CI 95% 2.46–14.37), and calls occurring in British Columbia (B.C) (OR 3.55, CI 95% 1.46–7.33) had a significantly higher likelihood of a drug poisoning. No deaths were recorded and only 3 false callouts had occurred. The overall drug poisoning event incidence to phone calls was 1.2%.

**Conclusion:**

NORS presents a complimentary opportunity to access harm reduction services for individuals that prefer to use alone or face barriers to accessing in-person supervised consumption services especially gender minorities with high-risk use patterns.

**Supplementary Information:**

The online version contains supplementary material available at 10.1186/s12889-023-16751-z.

## Introduction

The drug poisoning epidemic grew exponentially over the course of the pandemic in Canada and continues to result in harm to individuals and communities [1]. An effective solution to mitigate the harms associated with an increasingly toxic drug supply has been the use of supervised consumption sites (SCS) which provide safer spaces for individuals to use substances through access to individuals trained to respond in the event of an acute drug poisoning [2–4]. SCS is a broad term, and includes other types of facilities (which are similar in scope but not necessarily identical) such as “drug consumption rooms”, “safe injection facilities” (SIFs), or overdose prevention sites (OPS). Legally, within Canada, SCS’ must obtain a legislative exemption allowing substance users to bring in illicit substances and use them within these facilities without penalty or fear of criminalization. These sites improve access to care, prevent transmission of blood-borne illnesses and provide a protective space away from violence and criminalization for people to use their substances [5]. Despite these successes, multiple interrelated factors underlie challenges in accessing SCS including rurality and geographic barriers, stigma and political will, personal comfort and safety of using alone, costs and staffing supports, and unsubstantiated claims of increased crime and social disorder [5]. The COVID-19 pandemic only exacerbated the lack of access to SCSs, where many facilities were briefly closed in Canada thereby limiting the ability of these sites to safely provide support [6].

Fortunately, novel virtual overdose monitoring services (VOMS), or mobile overdose response services (MORS), have been developed as an adjunct solution to SCS, aiming to address the current gaps in harm reduction care for these individuals. These technology-based overdose prevention services include a variety of hotline, mobile phone applications, and automated services which can virtually provide harm reduction support for individuals using alone [7].

Since December 2020, Canadians have been able to use the National Overdose Response Service (NORS) which offers access to harm reduction and social support via a telephone line operated by persons with lived and living experience of substance use, henceforth peers, who provide virtual monitoring during a substance use session [8]. Peer operators can activate the client's preferred emergency response including contacting nearby friends or family members or emergency medical services (EMS) during an overdose event [8]. The design and implementation of the NORS service have been previously described in Matskiv et al. 2022 and Ritchie & Ghosh 2022 [9, 10]. Briefly, the aims of NORS are 1. To provide individuals with telephone-based supervision support to all Canadians that use substances. 2. To mitigate the risk of acute drug overdose/poisoning deaths by notifying community-based supports and 3. Facilitate timely access to additional medical and psychosocial services as needed 4. Virtually extend and improve the reach of physical SCSs and OPS as well as other harm reduction services to those who need them [8].

These various services have the potential to improve access to harm reduction resources, however, they still face unique challenges to their implementation and acceptance [11]. Despite these barriers, and building upon a heightened interest in information communication technology in healthcare, the Covid-19 pandemic has bolstered uptake of these services, particularly in areas focusing on preventing substance use-related mortality [9]. This retrospective observational study aims to 1. describe the service utilisation characteristics of the NORS system and 2. identify attributes associated with an overdose requiring medical intervention. These findings will provide valuable information about those who utilise the service and service use outcomes.

## Methods

This retrospective study used data gathered from two complementary sources. One source was from a system known as “Talkroute” which automatically records information of each call, including the time of call and length of the call, irrespective of call type. An additional source of information was NORS call logs, which are manually entered by operators of the line to track each call made. “Talkroute” data is helpful in that it tracks any phone call coming into NORS, however, is limited in the information it provides, whereas NORS call logs are limited in that they are manually entered by operators and are sometimes forgotten, or missed by staff. Both sources of information help clarify overall call volume, length, and timing.

All this information was collected with the primary purpose of informing the NORS program, funders, and stakeholders from December 2020 to April 2023. Due to privacy concerns, gender and Indigenous identity data gathering began through voluntary disclosure in July 2022 and information was retrospectively applied to previous callers identified through an anonymous unique user identification system. This system involves generating a unique use code based on an individual's disclosed first name, last name, and date of birth. All callers who voluntarily disclose additional information had these retrospectively applied to calls that reported the same unique client I.D. The analyses included all completed calls to the service. The time of call is automatically recorded when operators complete the electronic call log. For purposes of our analysis, we excluded calls that were above or below 2 standard deviations of call length time. For purposes of our analysis, we excluded calls that were above or below 2 standard deviations of call length time. Calls outside this range were considered non-representative of the true completed calls and likely represented, hang ups, wrong numbers, prank/ abusive calls, technical issues or other highly unusual calls.

All demographic variables including gender, age, language, route, and substance of use were voluntarily and optionally disclosed. Callers are required to provide their location in order to facilitate rescues, but for privacy, the NORS operators only record the town and province. All adverse events were recorded including mental health crises and acute drug overdoses.

**Fig. 1:**
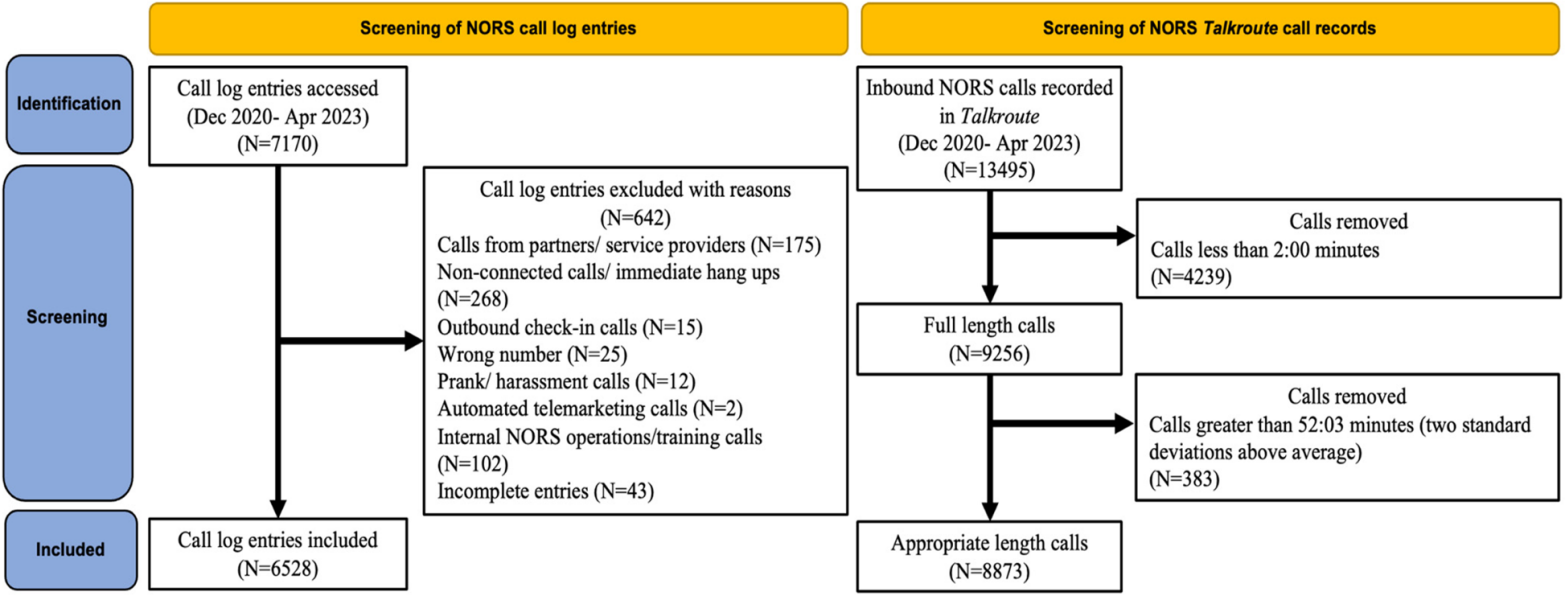
Flow chart of NORS call log entries and Talkroute call records *The NORS operator call log and Talkroute call records report a wide difference in call volume most likely due to differences in recording of completed calls. Operators may only record one call if calls are dropped and the connection was subsequently re-established. Technical issues with the call log, operators forgetting to record or confusion about which type of calls should be recorded are likely smaller contributing factors

Adverse events were defined as any event in which the NORS operators believed the person was in danger (or a danger to others) which required some kind of verbal mediation, a physical or in-person response, or an emergency transfer to another service. Referrals to healthcare services were also recorded. For this analysis, we only included adverse events involving acute drug poisoning (overdose) which led to an in-person rescue attempt by EMS or community members with naloxone. Call information is depicted in Fig. 1. For all overdose events, operators followed up with callers to provide support and verify outcomes.

### Statistical analysis

To examine our key outcome of overdose risk, odds ratios with 95% confidence intervals were calculated for all key indicators of interest: self-identified gender, size of the community, province, substance of choice, route of substance consumption, and time of call. In addition to odds ratios, two-tailed Chi-square tests at 5% level of significance were used to identify significant associations between drug poisoning events and our key indicators. Where counts within a group were less than 5, Fisher's exact test was used. Key Indicators were all organised into binary variables (“yes” and “no”). Missing values were coded as “N/A” and excluded from the calculations. The level “no” was used as the reference category for each factor. Odds ratios compared the odds of overdose events between each factor level within the factor’s sample. Only key indicators with a factor level containing 2 or more overdose events had odds ratios calculated. Odds ratios were calculated following traditional methods [12]. Descriptive statistics were run with SPSS software Version 28 [13]; odds ratios and a forest plot were created using R version 4.1.2 [14].

All findings and the methodological approach were reviewed with people with lived experiences with substances who provided feedback on the manuscript and interpretation of findings. Ethics approval was obtained from the University of Calgary (REB21-1966). Where possible, the STROBE checklist was utilised to help guide the evaluation. The research methodology for this study was not preregistered and the results should be considered exploratory.

## Results

### Demographics and client characteristics

The sample includes a total of 6528 completed calls from the NORS call logs and 8873 total call records from the call management software *Talkroute*. Call exclusions can be seen in Fig. 1.

**Fig. 2:**
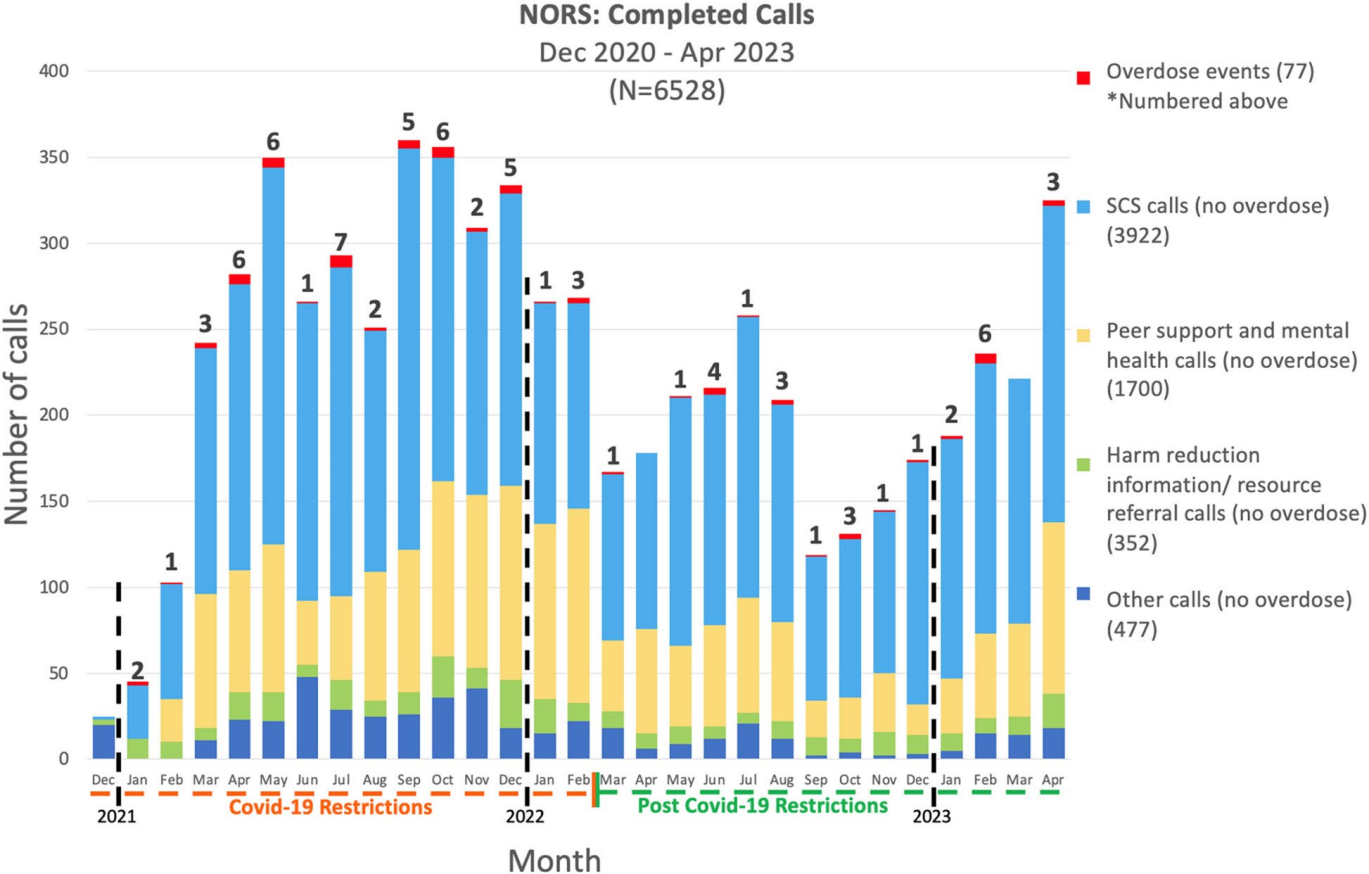
NORS completed calls between December 2020 to April 2023

Of the recorded calls in the operator call log, 3994 (61.2%) were for SCS, 1703 (26.1%) were primarily for peer support conversations/ mental health support, 354 (5.42%) were for information about the service, harm reduction education, or to obtain resources, and 477 (7.31%) were for other purposes. A total of 455 unique callers contacted the line, with an average of 13.5 calls per client (STD + _108.1), with 333 unique callers using the SCS features with an average of 11.9 calls each (STD + _ 70.4). Extrapolating current self-reported gender data to previous callers, 3235 (49.6% and 61.0 average calls per unique user) of calls were from women, 897 (13.7% and 19.2 average calls per unique user) from men and 1070 (16.3% and 212.8 average calls per unique user) were from gender-diverse individuals and the remaining 1326 (20.3%) did not disclose their gender. Additionally, 409 (6.26%) calls were from individuals reporting Indigenous identity. Descriptive statistics indicating the primary reason behind individuals’ calls are shown in Fig. 2. See Table 1 for a summary of client and service utilization characteristics. Additional figures of NORS trends and uptake can be found in the supplementary materials (see Additional file 1).

**Table 1:** Chi-Square and fisher’s exact results of overdose events by caller characteristics

**Characteristic**	**Total Calls (%)**	**Overdose events (%)**	**Chi-Square/ Fisher’s** **Exact(**†)	***P*** **-value**
**Total Calls**	**6528**	**77 (100)**		
**Gender**	**6528**			
Men	897 (13.7)	4 (5.2)	.029*†	.029†
Women	3235 (49.5)	22 (28.5)	13.73**	< .001
Gender Diverse	1070 (16.3)	22 (28.5)	8.44*	.004
Not disclosed	1326 (20.3)	29 (37.6)	14.49**	< .001
**Age**	**6528**			
Under 18	45 (0.69)	0 (0)	1†	1.00†
18 -30	3043 (46.6)	30 (39.0)	1.83	.176
31 -40	427 (6.5)	4 (5.2)	.23	.631
41 -50	2225 (34.1)	31 (40.3)	1.32	.250
51 -60	305 (4.7)	4 (5.2)	.78†	.827
60 +	17 (0.26)	0 (0)	1†	.652
Missing	466 (7.1)	8 (10.4)	1.24	.265
**Indigenous Identity**	409 (6.26)	1 (1.3)	0.092†	.070
**Region**	**6528**			
British Columbia	187 (2.86)	7 (9.1)	10.86**	< .001
Prairie (AB, SK, MB)	1108 (16.9)	7 (9.1)	3.44	.064
Ontario	4137 (63.3)	56 (72.7)	2.94	.087
Quebec	673 (10.3)	0 (0)	< .001**†	< .001†
Atlantic Canada: (NS, NB, NL, PE)	38 (0.58)	2 (2.6)	0.074†	0.074†
Northern Territories (YT, NT, NU)	20 (0.30)	1 (1.3)	0.21†	0.212†
Other regions	30 (0.45)	1 (1.3)	0.30†	0.300†
Unknown	335 (5.13)	3 (3.9)	0.80†	0.798†
**Community Type (population)**	**6528**			
Urban (> 100,000)	5796 (88.7)	68 (88.3)	0.018	.894
Medium (10,000—100,000)	161 (2.46)	5 (6.5)	5.25*	.041
Rural (< 10,000)	34 (0.52)	1 (1.3)	0.33†	0.333†
Unknown	537 (8.22)	3 (3.9)	0.21†	0.210†
**Time of Call**	**6528**			
00:00 to 06:00	574 (8.79)	12 (15.5)	4.481*	.035
06:00–11:59	1532 (23.4)	11 (14.2)	3.66	.056
12:00–17:59	2152 (32.9)	21 (27.2)	1.14	.285
18:00—23:59	2270 (34.7)	33 (42.8)	2.25	.134
**Day of the Week**	**6528**			
Sunday	795 (12.2)	6 (7.8)	1.40	.236
Monday	975 (14.9)	11 (14.3)	.026	.872
Tuesday	938 (14.4)	9 (11.7)	.46	872
Wednesday	979 (15.0)	19 (24.7)	5.73*	.017
Thursday	965 (14.8)	8 (10.4)	1.19	.275
Friday	941 (14.4)	10 (13.0)	.13	.720
Saturday	935 (14.3)	14 (18.2)	.95	.331
**Season and COVID-19**	**6528**			
Spring (Mar 20- Jun 20)	1884 (28.8)	22 (28.5)	0.003	.955
Summer (Jun 21- Sep 22)	1526 (23.3)	20 (25.9)	0.29	.588
Fall (Sep 23- Dec 20)	1405 (21.5)	16 (20.7)	0.025	.873
Winter (Dec 21- Mar 19)	1713 (26.2)	19 (24.6)	0.099	.753
COVID-19 restrictions (Mar 1, 2020—Mar 1 2022)	3917 (60.0)	51 (66.2)	1.26	.262
**Call Type**	**6528**			
Supervised Consumption	3994 (61.1)	72 (93.5)	34.28**	< .001
Mental Health	1703 (26.0)	3 (3.9)	< .001**†	< .001
Info and Resources	354 (5.42)	2 (2.6)	0.44†	.442
Other / Unspecified calls	477 (7.30)	0 (0)	.006*†	.006
**Substance Used**	**3970 events with 4120 substances**			
Opioids	3088 (77.7)	66 (85.7)	46.12**	< .001
Cocaine	516 (13.0)	4 (5.2)	0.52†	.523†
Methamphetamine* (Overdose events only, not psychosis or other adverse events)	290 (7.30)	10 (12.9)	13.40**	< .001
Depressants (e.g. Benzodiazepines and Alcohol)	38 (0.96)	1 (1.3)	0.36†	.364†
Other	32 (0.81)	0 (0)	1†	1.00†
Unknown	156 (3.92)	3 (3.9)	0.43†	0.433†
Polysubstance Use	157 (3.95)	10 (12.9)	37.17**	< .001
Opioids and Methamphetamines	52 (1.31)	5 (6.5)	32.00**	< .001
Opioids and Depressants	10 (0.25)	0 (0)	1†	1.00†
Cocaine and Methamphetamines	7 (0.18)	0 (0)	1†	1.00†
**Route**	**4183 events using 4276 Routes**			
Injection	2265 (54.1)	39 (50.6)	8.75*	.003
Smoking	1299 (31.1)	32 (41.5)	22.93**	< .001
Insufflation / Snorting	129 (3.08)	2 (2.6)	0.67†	.0.665†
Oral	213 (5.09)	0 (0)	0.18†	0.183†
Other	11 (0.26)	0 (0)	1†	1†
Unknown	359 (8.58)	5 (6.5)	0.15	.700
Poly Route	90 (2.15)	6 (7.8)	23.57**	< .001

*P*-value: < 0.01 “*”, < 0.001 “**”, fisher exact text †

The NORS operators logged a total of 173 adverse events on the line, with the majority being drug poisoning events (113, 65.3%). The remaining events were comprised of mental health emergencies, including suicide prevention (44, 25.4%), domestic violence interventions (9, 5.20%), and helping redirect mistaken individuals to the correct poison control service for poisonings (7, 4.05%). For our analysis, we only included the 77 (44.5%) overdose events that resulted in interventions from either EMS (65, 37.6%) or a designated contact (12, 6.93%). While not included in this paper, six (3.47%) of mental health emergencies also required EMS or a designated contact. The excluded 36 drug poisoning events were either handled by breathing coaching via NORS staff (24, 13.9%), transferred to another crisis line (10, 5.78%), or the response type was unrecorded (2, 1.16%). A further description of NORS adverse events is included in the supplementary materials (see Additional file 2).

**Fig. 3:**
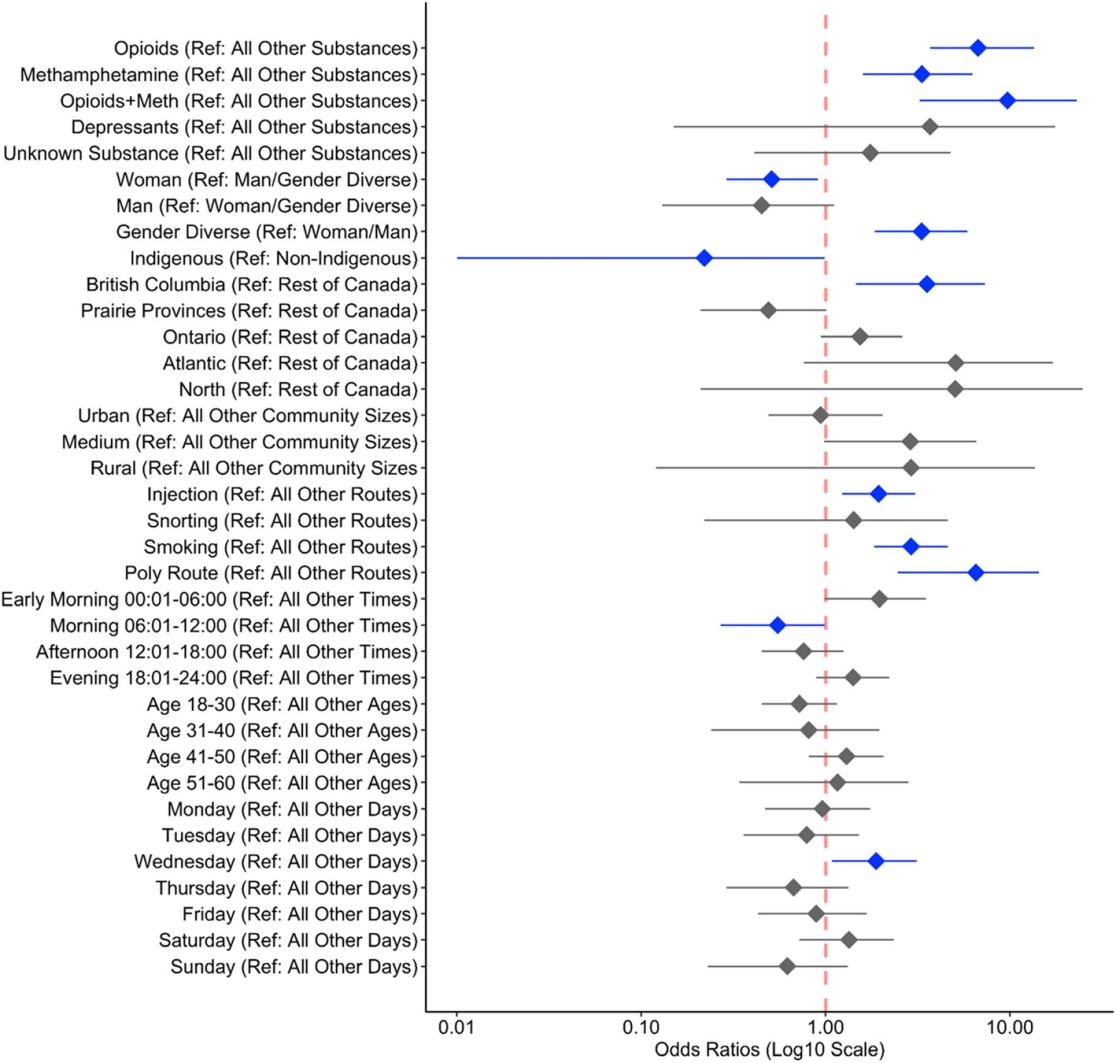
The likelihood of a NORS caller experiencing an overdose/ drug poisoning where blue indicates statistically significant results

Odds ratios were established for the key predictors. We found NORS callers using opioids, methamphetamine, and the two substances in combination had significantly increased odds of experiencing an overdose compared to other substances (Table 2, Fig. 3). In addition, gender-diverse callers had significantly higher odds of experiencing an overdose compared to self-identified men and women, with women having a decreased risk of overdose (Table 2, Fig. 3). Indigenous individuals who were using the line also experienced lower odds of an overdose event, however, our confidence interval is large due to a relatively small sample size (Table 2, Fig. 3). Although most calls originated in Ontario, the likelihood of a call resulting in an overdose was significantly higher in B.C (Table 2, Fig. 3). Callers that injected, smoked, or used multiple routes to use their substances had a higher chance of experiencing an overdose (Table 2, Fig. 3). There were no differences in the size of the community in regards to the risk of overdose. COVID-19 did not impact the risk of overdose. Lastly, we found overdoses had a significantly lower likelihood of occurring during the hours of 06:00–12:00 compared to all other times. Caller age was not a significant factor in the likelihood of overdosing in comparison to the entire sample.

**Table 2:** The likelihood of a NORS caller experiencing an overdose/ drug poisoning

Characteristic	Total Sample (Prob. of Overdose %)	Odds Ratios (95% CI)
Substance (Ref: All other substances)	3970	
Opioids	(2.1)	6.72 (3.69–13.52)***
Methamphetamine	(3.4)	3.33 (1.59—6.26)***
Opioids and Methamphetamine	(9.6)	9.70 (3.24—23.06)***
Depressants	(2.6)	3.69 (0.15—17.62)
Unknown substance	(1.9)	1.75 (0.41—4.76)
Gender	5202	
Woman (Ref: Man)	(0.4)	0.51 (0.29—0.91)*
Man (Ref: Woman/Gender Diverse)	(0.6)	0.45 (0.13—1.11)
Gender Diverse (Ref: Woman/Man)	(2.0)	3.32 (1.85—5.89)***
Indigenous Identity (Ref: Non-Indigenous)	6528	
Indigenous	(0.2)	0.22 (0.01—0.99)*
Province (Ref: Rest of Canada)	6163	
British Columbia	(3.7)	3.55 (1.46—7.33)*
Prairie Provinces	(0.6)	0.49 (0.21—1.01)
Ontario	(1.3)	1.54 (0.94—2.61)
Atlantic	(5.2)	5.09 (0.76—17.11)
Northern Canada	(5.0)	5.05 (0.21—24.77)
Community Size (Ref: Medium/Rural)	5991	
Urban	(1.1)	0.94 (0.49—2.04)
Medium	(3.1)	2.88 (0.98—6.57)
Rural	(2.9)	2.91 (0.12—13.67)
Route of Consumption (Ref: All other routes)	4183	
Injection	(1.7)	1.94 (1.23—3.06)***
Insufflation/ Snorting	(1.5)	1.42 (0.22—4.59)
Smoking	(2.4)	2.91 (1.83—4.59)***
Poly Route	(6.7)	6.54 (2.46—14.37)***
Time of Call (Ref: All other times)	6528	
00:00–06:00	(2.0)	1.96 (0.99—3.51)
06:00–12:00	(0.7)	0.55 (0.27—0.99)*
12:00–18:00	(0.9)	0.76 (0.45—1.25)
18:00–24:00	(1.4)	1.41 (0.89—2.22)
Age of Caller (Ref: All other ages)	6000	
18–30	(0.9)	0.72 (0.45—1.15)
31–40	(0.9)	0.81 (0.24—1.96)
41–50	(1.3)	1.30 (0.81—2.06)
51–60	(1.3)	1.16 (0.34—2.82)
Weekday (Ref: All other weekdays)	6528	
Monday	(0.7)	0.96 (0.47—1.75)
Tuesday	(1.1)	0.79 (0.36—1.52)
Wednesday	(0.9)	1.88 (1.08—3.12)*
Thursday	(1.9)	0.67 (0.29—1.33)
Friday	(0.8)	0.89 (0.43—1.67)
Saturday	(1.0)	1.34 (0.72—2.34)
Sunday	(1.4)	0.62 (0.23—1.32)

*P*-value: < 0.05 “*”, < 0.01 “**”, < 0.001 “***”

## Discussion

In this retrospective observational study of NORS calls we found that the clients accessing the services mostly identified as women and were from large urban centers in Ontario, calling for overdose monitoring for opioid use through smoking or injecting routes. To date, there have been no fatal events, and as such virtual overdose services like NORS present a viable harm reduction measure, particularly for gender minorities. This option provides a low-risk, low-barrier, safe space for harm reduction and management of substance use. To the best of our knowledge, this is the first study to characterize the service utilization patterns of any virtual overdose monitoring /prevention service, as well as examine predictive factors of overdose for these services.

### Service utilisation characteristics

#### Gender

Call-logs from the NORS phone line indicate that a majority of calls (where the client disclosed their gender identity) were either women or gender diverse (82.8%, *n* = 4305). This finding is similar to previous research on mental health hotlines which has found greater utilization among women [15]. Previous studies have found that women are less likely to express a willingness to use in-person SCS than men [16]; therefore the use of VOMS may reduce barriers to service access for these populations [17]. One explanation behind this phenomenon is that while SCS’ and OPS’ provided a sense of safety, they are traditionally considered masculine spaces where women routinely were subjected to harassment. A qualitative study found that routine harassment within OPS’ were tolerated as the alternative to using alone was considered more risky [18]. NORS, and other similar services such as Never Use Alone in the United States, BRAVE, and automatic overdose monitoring services like Connect by Lifeguard and The Digital Overdose Response Service (DORS) may provide a sense of overdose safety for gender minorities in light of potential violence and harassment at physical sites [11].

In a study of one in-person SCS, 81.6% of those who became unresponsive due to a witnessed opioid overdose were men [19]. This contrasts the 44 overdoses (57.1%) that were from women and gender-diverse callers of the NORS line, with men accounting for only four (5.2%). The calculated odds ratios of gender and likelihood of overdose found that women were significantly less likely to have an overdose event (0.51 (0.29–0.91)), and gender-diverse people were significantly more likely to have an overdose event (3.32 (1.85–5.89)). Through the results of our analysis, NORS presents as an especially useful harm reduction option for gender minorities who may not be accessing SCS services otherwise.

#### Indigeneity

Relatively few individuals interacting with NORS disclosed their Indigenous identity (409, 6.26%) but it was found to be a significant predictor of not having an overdose event (OR 0.22, CI 95% 0.01–0.99). In-person SCS reports a much larger percentage of individuals using these services as Indigenous identifying (32–33% compared to our studied 6.26%) [20]. Previous studies indicate that mobile phone access does not differ significantly between populations of people who use substances accessing SCS (48%(*n* = 212)) [21] as well as young Indigenous individuals who have used substances in Vancouver, B.C (45% (*n* = 59)) [22]. The Indigenous community continues to be disproportionately impacted by the drug poisoning epidemic representing 15% of drug poisoning/ overdose mortality despite representing 6% of the population in B.C [23, 24]. Extending the outreach of harm reduction services to Indigenous communities through partnerships with these communities, such as those seen in the Connect app by LifeGuard [25], should be prioritized to address the disparities of care facing this population. More targeting and culturally responsive advertising of NORS may assist in outreach to this population. A strategy currently being explored at NORS to improve equitable access to Indigenous communities is to provide services in local Indigenous languages and employ Indigenous operators. Indigenous peers operating the line could improve connectivity with Indigenous communities and improve utilization and uptake within this population.

#### Geographic factors

Most NORS calls originated in the province of Ontario. Ontario is one of the most populated regions of Canada, and the number of callers is proportional to the distribution of the Canadian population. It was felt that many of these calls were also due to the strong presence of NORS staff and leadership in Ontario, which helped with networking and word-of-mouth communication with local substance-using and harm-reduction communities.

Although most calls originated in Ontario, we found that a higher proportion of calls resulting in overdose interventions occurred in B.C. These findings are consistent with current trends in drug use in Canada, with B.C. having an illicit drug overdose mortality rate nearly double that of the Canadian average [26], with even higher rates in their northern rural areas [27]. In our analysis of overdose, while using NORS, the probability of overdose was 3.7% in B.C. Furthermore, recent Canadian mortality statistics describe B.C. as having more than twice the overdose mortality rate compared to the national average (42.1 versus 19.0 deaths per 100,000) [26]. This is partially attributed to the toxicity of the drug supply in the province [27].

While rural populations only comprised a small proportion of callers, our study demonstrates the feasibility of NORS as a strategy to provide harm reduction services in these areas and reduce health inequalities for remote and rural populations. Continued outreach efforts and dissemination of harm reduction strategies are needed to address these health inequalities and higher rates of fatal drug overdose seen in these areas [27]. The penetration of naloxone kits within various small communities in Canada has been seen as a potential marketing option for virtual services and is already being utilized by services like DORS in Alberta [28].

#### Time, Seasonal variation, and COVID-19

From our analysis, call volume was consistent throughout the day barring the early hours of 0:00 to 6:00. It was previously hypothesised that the line would be used more in late hours as some SCSs in Canada have limited times of operation. Interpretation of results should consider that call times were recorded at the time when staff submitted a call-log entry and therefore may not be representative of actual call volume. Of note, overnight calls also had a higher likelihood of an overdose occurring at a rate of 2.0% from 2400 to 0600. Although these rates are modest, they are substantive in comparison to the probability of an overdose for all calls at 1.2%. Findings from our data are comparable to those of a study on EMS calls for suspected drug overdoses in Baltimore, U.S. which records a decrease in call volume during the hours 6:00–7:00 AM [29]. The only statistically significant time trends observed were that overdoses were less likely to occur between 6:00 and 12:00. These results can potentially help inform staffing decisions for the establishment of other VOMS ensuring that staff are available during busy periods.

When examining overdose events by the day of the week, we found drug overdoses to be more likely to occur on Wednesdays. Currently, we have no clear reason for this trend, but we hypothesize that it could be related to the timing of social assistance payments, such as the phenomena in B.C. where drug-related adverse events increase on the payment dates. Of note the payment dates for B.C. are Wednesdays but this is not universal throughout the country [30].

It was previously hypothesized that the line would be utilized more in colder months when individuals were more likely indoors and not likely to go to a physical SCS, however, our results demonstrate no seasonal variation in utilisation. Indeed, this is in contrast to findings from a previous qualitative study that found that individuals favoured the convenience of virtual overdose services like NORS, especially in cold weather [31]. Lastly, the line showed a decrease in overall utilisation post-pandemic, however, there was no indication that the pandemic period predicted an increased risk of overdose.

#### Call type

Most calls to the line were for SCS comprising 61.1% of the call volume. One pertinent finding was the high percentage (26.0%) of mental health support and crisis management calls that were seen on the line. Previous United States population-based research on individuals with substance use disorders indicated that 43% have associated symptoms of mental illness [32]. Peer support has recently emerged as a viable strategy for improving engagement and outcomes among clients in both the mental health and substance use fields [33, 34], and having support for individuals who both use substances and have concurrent mental health concerns is an important gap addressed by NORS. Hotlines like NORS and Never Use Alone have strongly incorporated peer support into their services, noting that peers are key in creating viable trusting spaces for substance users, removing barriers to access services, and improving acceptability by reducing stress and re-traumatization of clients [33].

#### Substance used and route of use

The vast majority of substances used on the line were opioids and methamphetamines. Interestingly, methamphetamine use as a solo substance was associated with a higher risk of overdose, a surprising result reflective of the potential cross-contamination of substances. Indeed, there are rising rates of fentanyl contamination in methamphetamines, with advocates strongly suggesting the utilization of fentanyl test strips to avoid potential overdose events with methamphetamines [35]. Virtual overdose monitoring services / mobile overdose response services like NORS may provide an additional harm reduction tool to avoid overdose events from contaminated methamphetamines. Polysubstance-based overdoses were also predictive of overdose events on NORS, similar to trends seen in the community [36].

As the service is primarily a drug overdose prevention service, many callers were utilizing opioids on the line with primary routes of substance use being either injection (54.1%) or inhalation (31.1%), with both of these routes being predictive of potential overdoses on the line. Inhalation however makes up the majority (69%) of individuals’ preferred method to use substances within B.C, Canada [37]. Unfortunately, only a minority of physical SCS provide inhalational access for substance use [38]. Substance users describe the need for increased supervised inhalational support citing the need to smoke indoors during poor weather conditions, but also to smoke in privacy and out of the public eye [39]. From a health equity lens, there is a large disparity in providing inhalational supervised consumption services to groups of substance users, a serious gap that needs further intervention and support. Virtual services like NORS may therefore present a viable option for individuals who may be unable to benefit from in-person services.

#### Outcomes

The rate of overdose virtually witnessed on the NORS line are slightly higher than findings reported from Health Canada SCSs between 2017–2023 which indicate 47,000 overdoses over 4,170,000 SCS visits resulting in an overdose rate of 0.0112 [40]. NORS had a slightly higher rate at 0.012 (77 overdoses per 6528 uses). In recent years, overdose mortality rates have doubled in Canada and likely provide a partial explanation for the higher overdose rates seen within our study [26].

Response times have been criticized as a limitation of VOMS/ MORS [41]. While recognizing the relatively small sample size afforded by the data, to date there have been no fatalities recorded on the NORS line. While a more robust dataset would be required to draw more substantive conclusions, these findings contrast the opinions of both people who use substances and policymakers on the effectiveness of these services in preventing overdose death [17]. Emergency response planning, which includes the ability for timely response in the event of an emergency, such as those previously described, will be integral to the continued success of the service [10].

NORS clients usually used EMS as part of their emergency response plan. Operators called EMS in 84.4% (*n* = 65) of overdose events and activated a community-based response 15.5% (*n* = 15) of the time. Furthermore, EMS was called out for an additional four mental health events. Anecdotally there continue to be voiced concerns, particularly from first responders, that rates of false call-outs may be high and cause increased demand and stress on services however only three (3.90%) false call-outs occurred over 29 months. This is particularly important as the rates of inaccurate callouts and false positives were low, in comparison to other emergency dispatch technologies such as personal alarms for falls, that report a rate of 13% of false calls [42]. These numbers may differ for more automatic nonoperator-led harm reduction technology such as the LifeGuard, iKeepr, or DORS app [25].

### Limitations

This study is an initial overview of calls to a VOMS/MORS hotline. The low rate of overdoses impacted the ability to examine and interpret findings for specific subpopulations. This also impacted the extent of the statistical analyses and statistical power. Collinearity was also not evaluated which may undermine the statistical significance of our results. Secondly, data collection was conducted both retrospectively and upon completion of substance use and monitoring sessions which presented recall biases and potential logging errors. Third, our data relied on self-reported information in order to preserve client privacy, which makes the number of unique participants, their gender, and indigenous identity subject to reporting bias and missing information. Efforts were made in the analysis to consider these potential biases as well as inform our own recording practices. As NORS is an ongoing crisis service, the administrative data presented challenges with missing values and recall bias. Of note, only calls which resulted in overdose events were subject to follow-up to confirm details of the health of the client.

Further, NORS is only one type of VOMS/MORS, with numerous other types existing including Never Use Alone, Lifeguard, iKeepr, Brave, and Canary, to name a few. The results presented from NORS, being a peer-to-peer hotline may not necessarily extend to the more automated services like Lifeguard and DORS.

## Conclusion

The findings of our analysis indicate that a low-barrier VOMS/MORS like NORS is an additional tool to potentially reduce the harm associated with substance use for a variety of populations with diversity in substance use patterns, preferences, and demographic and geographic differences. Overall findings from our analysis suggest NORS provides a complimentary option to current harm reduction services, particularly for women and gender non-binary individuals. Also, accessing crisis support for mental health symptoms appeared to be an important secondary benefit of NORS. The results of this study can be used to help guide policymakers and public health officials in resource allocation and the establishment of similar services in the future. Similarly, this can help clinicians to make informed recommendations about harm reduction services for patients who use drugs, especially for hard-to-reach and under-serviced groups who do not have access to physical SCS sites or cannot access existing harm reduction services due to individual and structural barriers. Future research is needed on the economic impact of these services and the health, and quality of life benefits that arise from engagement with these services. Future research should also examine the perspectives and experiences of Indigenous groups, women, and gender-diverse individuals, using these services and barriers to access for the broader population at risk of overdose.

### Supplementary Information

**Additional file 1.** Figures of NORS uptake over time.

**Additional file 2.** Description of adverse events recorded.

## Data Availability

The datasets generated and/or analysed during the current study are not publicly available due to privacy concerns around NORS clients’ illicit substance use but are available from the corresponding author on reasonable request. Supplementary figures describing the uptake and trends of NORS are available for free download in the supplementary files. Please cite all figures using the citation for this manuscript.

## Abbreviations

B.C: British Columbia
DORS: Digital Overdose Response System
EMS: Emergency medical services
NORS: The National Overdose Response Service
MORS: Mobile overdose response services
OPS: Overdose prevention sites
SCS: Supervised consumption sites
SIF: Safe injection facilities
VOMS: Virtual overdose monitoring services
