# Supplementary material for: Association of drug overdoses and user characteristics of Canada’s national mobile/virtual overdose response hotline: the National Overdose Response Service (NORS)
Source: BMC Public Health. 2023 Sep 27;23:1869. doi: 10.1186/s12889-023-16751-z (PMC10523711; doi:10.1186/s12889-023-16751-z)
Supplement: Supplementary file 1 — Additional file 1. Figures of NORS uptake over time. [file 12889_2023_16751_MOESM1_ESM.docx]

**Association of drug poisoning events/ overdoses and user characteristics of Canada’s national virtual overdose monitoring service: The National Overdose Response Service (NORS)**

**Supplementary files**

**Additional file 1:** Figures of NORS uptake over time
