# Supplementary material for: Association of drug overdoses and user characteristics of Canada’s national mobile/virtual overdose response hotline: the National Overdose Response Service (NORS)
Source: BMC Public Health. 2023 Sep 27;23:1869. doi: 10.1186/s12889-023-16751-z (PMC10523711; doi:10.1186/s12889-023-16751-z)
Supplement: Supplementary file 2 — Additional file 2. Description of adverse events recorded. [file 12889_2023_16751_MOESM2_ESM.docx]

**Additional file 2:** Description of adverse events recorded

| **Drug poisoning events included in overall analysis (77)** | **N (%)** |
| --- | --- |
| EMS (Primary) | 65 (84.4) |
| Designated contact    (Primary) | 12 (15.5) |
| **Assisted responses** |  |
| EMS + Designated contact | 3 (3.89) |
| EMS + Staff* | 4 (5.19) |
| **EMS: False Alarm** | 3 (3.89) |
| **Adverse events NOT included in analysis (96)** |  |
| Other drug poisoning    events | 36 (37.5) |
| Staff (Breath coaching) | 24 (25) |
| Crisis Line | 10 (10.4) |
| Unknown | 2 (2.08) |
| Mental health emergencies | 44 (45.8) |
| EMS | 4 (4.16) |
| Designated contact | 2 (2.08) |
| Staff (Mental health    first aid) | 29 (30.2) |
| Crisis line | 9 (9.37) |
| Other emergencies | 16 (16.6) |
| Domestic violence    assistance (staff) | 9 (9.37) |
| Poison control line    (Non-overdose event) | 7 (7.29) |
| *NORS staff stay on the phone and assist during all adverse event dispatches and thus this number is likely under reported. | |
